# Pyridine substituted BODIPYs: synthesis, characterization and cholinesterease, α-glucosidase inhibitory, DNA hydrolytic cleavage effects

**DOI:** 10.3906/kim-2105-69

**Published:** 2021-10-19

**Authors:** Burak BARUT, Hüseyin BAŞ, Zekeriya BIYIKLIOĞLU

**Affiliations:** 1 Department of Biochemistry, Karadeniz Technical University, Trabzon Turkey; 2 Department of Chemistry, Karadeniz Technical University, Trabzon Turkey

**Keywords:** Synthesis, BODIPY, anticholinesterase, DNA hydrolytic cleavage

## Abstract

In this study, the synthesis of new monostyryl (**BDPY-2**) and distyryl BODIPY dyes (**BDPY-4, BDPY-5**) containing pyridine groups has been reported for the first time. The acetylcholinesterase from *Electrophorus electricus* (AChE), butyrylcholinesterase from equine serum (BuChE), α-glucosidase from *Saccharomyces cerevisiae *and DNA hydrolytic cleavage actions of **BDPY-2**, **BDPY-4**, **BDPY-5** were investigated using various techniques. The results indicated that the compounds had varying inhibition properties against AChE, BuChE, and α-glucosidase. **BDPY-4** was the most potent compound on AChE with IC_50_ of 54.78 ± 4.51 µM, and Lineweaver–Burk plots indicated that the compound is bound to a site other than the active site as a noncompetitive inhibitor. The compound-protein binding experiment showed that **BDPY-4** changed the microenvironment around AChE. On the other hand, the compounds showed lower α-glucosidase inhibition than the positive control. The DNA hydrolytic cleavage effects were not observed on supercoiled plasmid DNA in the presence of the compounds as compared to negative controls. These findings suggested that **BDPY-4** might be a promising compound to treat Alzheimer’s diseases.

## 1. Introduction

Alzheimer’s disease (AD) is one of the major global health challenges among the elderly population and a chronic and progressive syndrome categorized by developing memory and perception impairment [1,2]. Nowadays, nearly 50 million people are affected by this illness worldwide, and there will be more than 120 million new cases estimated in 2050 [3,4]. The etiology of AD involves many pathways including low acetylcholine level, overproduction of the beta-amyloid peptide, hypoxia, reactive oxygen species, and tau protein phosphorylation [5]. Up till now, an effective therapeutic strategy is to boost acetylcholine levels in the brain by inhibiting cholinesterase enzymes (acetylcholinesterase (AChE), butyrylcholinesterase (BuChE)) and regulate acetylcholine in the human body [6]. To date, very few inhibitors are approved few drugs for AD treatment by the United States Food and Drug Administration (FDA) such as tacrine, galantamine, donepezil, but they impart several adverse effects such as hepatotoxicity, gastrointestinal disorders, and periphery side effects [5,7].

Diabetes mellitus (DM) is a chronic metabolic disorder caused by hyperglycemia with less insulin action or secretion or both [8]. DM triggers severe health complications including neuropathy, nephropathy, cancer, retinopathy, etc. Thus, the most effective strategy of DM treatment is to regulate bloodstream glucose levels control [9]. α-Glucosidase inhibitors have important roles to decrease glucose levels in the bloodstream by preventing the breakdown of carbohydrates into absorbable monosaccharides [10]. However, α-glucosidase inhibitors have several side effects such as gastrointestinal disorders, etc. [11]. Thus, it is crucial to seek for low side effect profile α-glucosidase inhibitors. 

4,4-Difluoro-4-bora-3a,4a-diaza-s-indacene (BODIPY), developed by Treibs and Kreuzer in 1968, displays a wide range of research fields such as material, medical research, diagnosis, and treatment due to their strong absorption, high fluorescence quantum efficiency, and stable chemical structure properties [12–14]. They have been used for variety of applications *viz*. as laser dyes, drug delivery, solar cells, fluorescent labels, anticancer agents in photodynamic therapy [15–22].

In medicinal chemistry, pyridine is a versatile heterocyclic nucleus finding applications. It is well-known that they have been exhibited various pharmacological and biological activities such as antiviral, anticancer, antidiabetic, anticonvulsant, anticholinesterase, antimicrobial, antiinflammatory, etc. [23–29]. Here, we aimed to define the biological activity of the **BDPY-2, BDPY-4**, and **BDPY-5** compounds on AChE, BuChE, α-glucosidase, and DNA.

## 2. Experimental

The materials, equipment, AChE, BuChE, α-glucosidase inhibitory, and DNA hydrolytic cleavage actions are given as supplementary information.

### 2.1. Synthesis

#### 2.1.1. 4-(3-Pyridin-4-ylpropoxy)benzaldehyde (1)

4-hydroxybenzaldehyde (1.57 g, 12.8 mmol), K_2_CO_3 _(3.53 g, 25.6 mmol), pyridine derivative (2 g, 12.8 mmol) were mixed in 20 mL dry DMF under nitrogen at 85 ºC for 24 h. The mixture was poured into ice-water and added 100 mL chloroform. Organic phase was dried with Na_2_SO_4_, and the crude product was performed to chromatograph on an aluminum oxide with chloroform as an eluent. Yield: 2.01 g (65%). IR (ATR), ν/cm^−1^: 3075 (Ar–H), 2955–2877 (Aliph. C–H), 1671 (C=O), 1592, 1507, 1469, 1425, 1397, 1311, 1255, 1214, 1151, 1110, 1018, 885, 799, 614. ^1^H NMR (400 MHz, DMSO-d*_6_*), (δ): 9.87 (s, 1H, =CH), 8.47 (d, 2H, ArH), 7.87 (d, 2H, ArH), 7.29 (d, 2H, ArH), 7.12 (d, 2H, ArH), 4.10 (t, 2H, CH_2_–O), 2.78 (t, 2H, Ar-CH_2_), 2.10-2.06 (m, 2H, -CH_2_-). ^13^C-NMR (DMSO-*d* *_6_*), (δ): 191.76, 163.96, 150.93, 149.80, 132.29, 130.10, 124.46, 115.38, 67.68, 31.15, 29.39. MALDI-TOF-MS m/z : 241.46 [M]^+^.

#### 2.1.2. BODIPY-2 (BDPY-2)

Compound (**1**) (250 mg g, 1.04 mmol), 2,4-dimethylpyrrole (0.23 mL, 2.08 mmol) and four drop of trifluoroacetic acid (TFA) were dissolved in dichloromethane (200 mL) stirred at rt for 24h. Then a solution of 2,3-dichloro-5,6-dicyano-1,4-benzoquinone (DDQ) (237 mg, 1.04 mmol) in CH_2_Cl_2_ (5 mL) was added slowly to the mixture. 3 mL triethyl amine (NEt_3_) was added. Then, 3 mL boron trifluoride diethyl etherate (BF3.OEt_2_) was added, and the reaction mixture stirred for 24h at rt. The mixture was washed with water, and the organic phase was dried over MgSO_4_; the solvent was evaporated under reduced pressure. The product was performed to chromatographed on an aluminum oxide column with a CH_2_Cl_2_:hexane (4:1) as solvent system. Yield: 261 mg (55%). IR (ATR) ν (cm^-1^): 3072 (Ar-H), 2923-2864 (Aliph. C-H), 1603, 1542, 1506, 1467, 1410, 1367, 1304, 1241, 1190, 1155, 1074, 972, 795. ^1^H-NMR (400 MHz, DMSO-d*_6_*), (δ): 8.45 (m, 2H, ArH), 7.28-7.25 (m, 2H, ArH), 7.10 (d, 2H, ArH), 6.89 (d, 2H, ArH), 6.17 (s, 2H, =CH), 4.04 (t, 2H, CH_2_-O), 3.91 (t, 2H, Ar-CH_2_-), 2.44 (s, 6H, CH_3_), 2.02-1.99 (m, 2H, -CH_2_-), 1.39 (s, 6H, CH_3_). ^13^C-NMR (DMSO-d*_6_*), (δ): 155.11, 150.83, 149.94, 143.20, 142.60, 136.32, 131.56, 129.57, 127.14, 124.41, 121.74, 115.65, 67.28, 66.97, 31.22, 29.54, 14.60. UV-Vis (CHCl_3_) λ_max _nm (log e): 503 (4.99). MALDI-TOF-MS m/z : 459.70 [M+H]^+^. 

#### 2.1.3. BODIPY-4 (BDPY-4)

BODIPY **2 **(100 mg, 0.21 mmol) and compound (**1**) (131 mg, 0.54 mmol) were dissolved in toluene (25 mL). Glacial acetic acid (0.3 mL, 3.57 mmol), piperidine (0.3 mL, 2.61 mmol), and a catalytic amount of magnesium perchlorate were added. The mixture was refluxed using Dean-Stark trap apparatus until was residuum. The product was performed to chromatographed on an aluminum oxide column with a CHCl_3_:benzene (3:2) as solvent system. Yield: 57 mg (30%). IR (ATR) *ν* (cm^−1^): 3067 (Ar–H), 2918–2849 (Aliph. C–H), 1597, 1537, 1509, 1485, 1461, 1385, 1242, 1199, 1161, 1107, 989, 823. ^1^H NMR (400 MHz, DMSO-d*_6_*), (*δ*): 8.51-8.46 (m, 8H, ArH), 7.58 (d, 4H, ArH), 7.40-7.36 (m, 8H, ArH), 7.13 (d, 4H, ArH), 7.04 (d, 2H, ArH), 6.94 (s, 2H, =CH), 6.82 (d, 2H, Ar–H), 4.07 (t, 6H, CH_2_-O), 2.89-2.84 (m, 6H, Ar-CH_2_), 2.14-2.11 (m- 6H, -CH_2_-), 1.47 (s, 6H, CH_3_). ^13^C NMR (DMSO-d*_6_*), (*δ*): 160.27, 160.03, 157.38, 155.31, 155.23, 155.07, 148.81, 147.62, 147.39, 147.16, 137.23, 137.12, 137.01, 136.94, 136.76, 130.11, 129.32, 129.06, 124.86, 115.62, 114.99, 67.30, 66.96, 31.37, 15.85. UV–Vis (CHCl_3_) *λ*_max_ nm (log *ε*): 644 (5.01), 591 (4.63), 370 (4.83). MALDI-TOF-MS m/z : 905.79 [M]^+^.

#### 2.1.4. BODIPY-5 (BDPY-5)

**BODIPY 5** was synthesized similarly to **BODIPY-4** by using compound (**3**) instead of compound (**1**). The product was purified by aluminum oxide column chromatography using chloroform as solvent. Yield: 123 mg (65%). IR (ATR) *ν* (cm^−1^): 3029 (Ar–H), 2919–2850 (Aliph. C–H), 1599, 1537, 1509, 1486, 1422, 1386, 1295, 1242, 1162, 1140, 1106, 1025, 990, 939, 826, 711. ^1^H NMR (400 MHz, DMSO-d*_6_*), (*δ*): 8.44-8.39 (m, 8H, ArH), 7.65 (d, 4H, ArH), 7.43 (s, 2H, ArH), 7.31-7.28 (m, 4H, ArH), 7.12 (d, 8H, ArH), 6.93 (s, 2H, =CH), 6.82 (d, 2H, ArH), 3.90 (t, 6H, CH_2_-O), 2.73 (t, 6H, Ar-CH_2_), 2.01-1.97 (m- 6H, -CH_2_-), 1.45 (s, 6H, CH_3_). ^13^C NMR (DMSO-d*_6_*), (*δ*): 160.11, 157.43, 152.48, 149.90, 147.63, 142.00, 138.72, 137.36, 136.97, 136.38, 133.32, 132.87, 130.08, 129.30, 126.65, 124.44, 123.95, 118.47, 116.39, 115.59, 114.98, 67.28, 66.93, 30.46, 14.85. UV–Vis (CHCl_3_) *λ*_max_ nm (log *ε*): 646 (5.04), 593 (4.62), 372 (4.86). MALDI-TOF-MS m/z : 906.00 [M+H]^+^. 

## 3. Results and discussion

### 3.1. Synthesis and characterization

**Figure 1:**
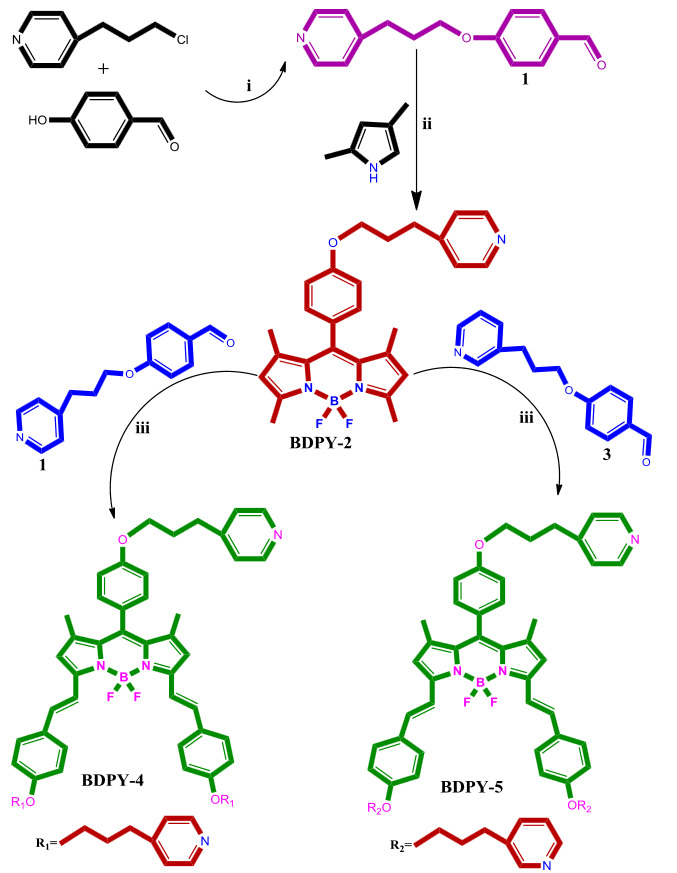
The synthesis of BODIPY dyes 2, 4 and 5. (i) K2CO3, 85 ºC, DMF. (ii) 2,4-dimethylpyrrole, DCM, TFA, DDQ, NEt3, BF3.OEt2. (iii) Glacial acetic acid, piperidine, Mg(ClO4)2, toluene.

The synthesis of monostyryl (**BDPY-2**), distyryl BODIPY dyes (**BDPY-4, BDPY-5**) containing pyridine groups are presented in Figure 1. Compound (**1**) was synthesized from the reaction of 4-(3-chloropropyl)pyridine with 4-hydroxybenzaldehyde in DMF. The monostyryl **BDPY-2 **was prepared by treating 2,4-dimethylpyrrole with 4-(3-pyridin-4-ylpropoxy)benzaldehyde in the presence of TFA, DDQ, NEt_3_, BF_3_.OEt_2 _in CH_2_Cl_2_. Then, distyryl BODIPY dyes (**BDPY-4, BDPY-5**) were synthesized using monostyryl **BDPY-2**, compound (**1**), compound (**3**) [20], piperidine, Mg(ClO_4_)_2_ as a catalyst in toluene at reflux temperature. 

**Figure 2:**
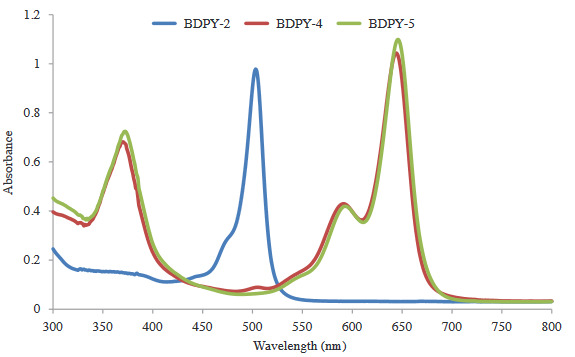
UV-Vis spectra of BDPY-2, BDPY-4, BDPY-5 in CHCl3.

**Figure 3:**
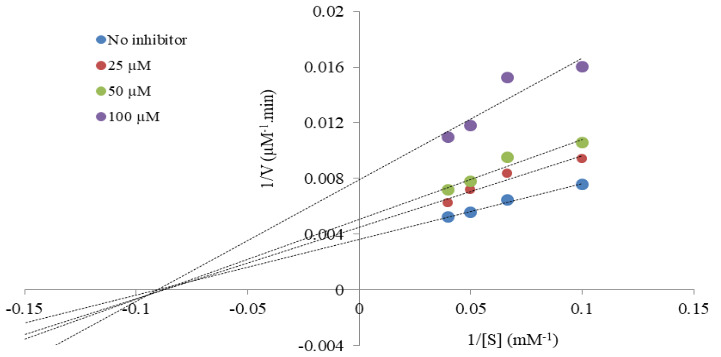
Lineweaver–Burk plot of BDPY-4 on AChE.

In the IR spectrum of (**1**), −C=O peak of (**1**) seemed at 1671 cm^−1^. In the ^1^H-NMR spectrum of (**1**), the aldehyde proton (=CH) resonated at 9.87 ppm. In the ^13^C-NMR spectrum of (**1**), the C=O group appeared at 191.76 ppm. The molecular ion peak of (**1**) was shown as 241.46 [M]^+^. In the IR spectrum of monostyryl **BDPY-2, **aldehyde peaks of (**1**) disappeared. In the ^1^H-NMR spectrum of monostyryl **BDPY-2, **aldehyde proton (=CH) vanished, and pyrrole =CH protons appeared at 6.17 ppm. The ^13^C-NMR data of **BDPY-2 **confirmed the structure. In the MALDI–TOF–MS of **BDPY-2**, the presence of the molecular ion peak at m/z = 459.70 [M+H]^+^ confirmed the structure. The IR spectra of distyryl BODIPY dyes (**BDPY-4, BDPY-5**) were similar with **BDPY-2**. In the ^1^H-NMR spectra of **BDPY-4, BDPY-5**, pyrrole =CH was observed at 6.94 ppm for **BDPY-4**, 6.93 ppm for **BDPY-5**. Also, The ^13^C-NMR data of **BDPY-4 **and** BDPY-5 **confirmed the structures. The molecular ion peaks were observed at m/z: 905.79 as [M]^+^ for **BDPY-4 **(Figure S1a), 906.00 as [M+H]^+^ for **BDPY-4 **(Figure S1b). The UV-Vis spectra of **BDPY-2, BDPY-4, BDPY-5 **were recorded in CHCl_3_ (Figure 2). As shown in Figure 3, **BDPY-2** showed an absorption peak at 503 nm, which is based on to a S_0_→S_1_ (p-p*) transition. Introduction of compound (**1**)**, **compound (**3**) to the **BDPY-2 **to give **BDPY-4, BDPY-5 **lead to red shifts (141 nm and 143 nm) in both the absorptions. **BDPY-4 **and** BDPY-5 **indicated absorption peaks at 644 nm and 646 nm.

### 3.2. AChE/BuChE inhibitory properties of the compounds 

The inhibition actions of the compounds (**BDPY-2, BDPY-4**, and **BDPY-5**) on AChE and BuChE were investigated according to our previously reported methods [30]. The results were expressed as IC_50_ and selective index (SI=BuChE/AChE) values. As shown in Table 1, the compounds showed dose-dependent inhibition on AChE and BuChE and IC_50_ values of the compounds ranged from 54.78 ± 4.51 to 184.87 ± 5.49 µM. **BDPY-4** was the most potent compound on AChE with an IC_50_ of 54.78 ± 4.51 µM. The IC_50_ values of **BDPY-2** and **BDPY-5** were determined as 72.46 ± 2.95 and 64.89 ± 3.89 µM, respectively against AChE. On the other hand, the IC_50_ values of **BDPY-2, BDPY-4**, and **BDPY-5** were 184.87 ± 5.49, 150.30 ± 6.09, and 170.30 ± 4.33 µM, respectively on BuChE. The SI values of **BDPY-2, BDPY-4**, and **BDPY-5** were 2.55, 2.74, and 2.62. The results showed that the compounds had lower anticholinesterase effects than galantamine as a positive control (IC_50_: 36.25 ± 0.58 µM for AChE; 65.32 ± 0.99 µM for BuChE), but they have a higher selective index (SI_galantamine_: 1.80).

**Table 1:** The IC50 (µM) and SI (BuChE/AChE) values of the compounds on AChE and BuChE.

	AChE	BuChE	SI
BDPY-2	72.46 ± 2.95	184.87 ± 5.49	2.55
BDPY-4	54.78 ± 4.51	150.30 ± 6.09	2.74
BDPY-5	64.89 ± 3.89	170.30 ± 4.33	2.62
Galantamine	36.25 ± 0.58	65.32 ± 0.99	1.80

**Figure 4:**
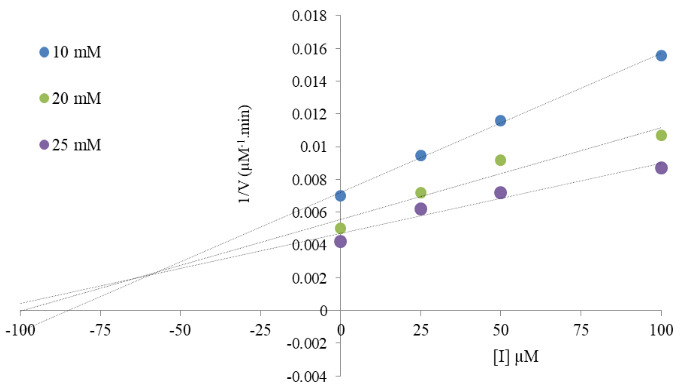
Dixon plot of BDPY-4 on AChE.

The mechanism for AChE inhibition was graphically determined by applying the Lineweaver–Burk and Dixon plots analysis of the most potent compound (**BDPY-4**). Acetylthiocholine iodide was used as a substrate for AChE inhibition. Lineweaver–Burk plot showed that *K* *_m_* (an index of the affinity of the enzyme for its substrate) was in similar values, but *V* *_max_* (maximal velocity of the reaction) decreased on increasing concentrations of the compound on AChE. While the *K* *_m_* value was 11.14 mM, the *V* *_max _* values changed from 277.78 µM/min to 126.58 µM/min (Figure 3, Table 2). The results indicated that BDPY-4 was a noncompetitive inhibitor and bound to a site other than the active site. On the other hand, BDPY-4 presented *K* *_i_* (inhibition constant) values of 57.20 ± 0.20 µM, according to the Dixon plot (Figure 4, Table 3). 

**Table 2:** The Vmax and Km values of the BDPY-4 on AChE.

	Vmax	Km
No inhibitor	277.78 µM/min	11.14 mM
25 µM	222.22 µM/min	11.14 mM
50 µM	196.08 µM/min	11.14 mM
100 µM	126.58 µM/min	11.14 mM

**Table 3:** The inhibitory type and Ki value of the BDPY-4 on AChE.

	Type	Ki
BDPY-4	noncompetitive	57.20 ± 0.20 µM

**Figure 5:**
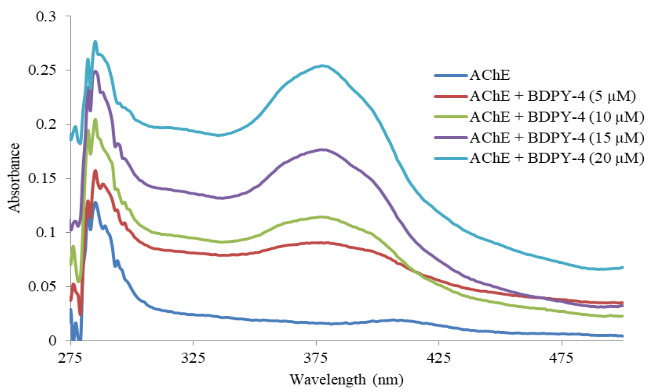
UV-Vis spectrum of BDPY-4 -AChE complexes.

To determine the structural change of AChE (10 µM) induced by **BDPY-4**, we measured the UV-Vis spectroscopy by adding the compound (5, 10, 15, and 20 µM) into AChE solution (10 µM). AChE has an absorption peak at 282 nm due to aromatic amino acids. As shown in Figure 5, the absorbance of the enzyme increased with various BDPY-4 concentrations (hyperchromism). In addition, the absorption peak shifted from 282 nm to 285 nm (redshift). The UV-Vis spectrum implies that BDPY-4 showed binding with enzyme and changed the microenvironment of some amino acid residues of AChE.

### 3.3. α-Glucosidase inhibitory properties of the compounds

The inhibitory properties of the compounds on α-glucosidase were investigated according to our previously reported methods [31]. The results were expressed as IC_50_ values. As shown in Table 4, IC_50_ values of the compounds ranged from 94.99 ± 4.77 to 218.62 ± 8.71 µM. **BDPY-4** was the highest α-glucosidase inhibitory effects among the tested compounds, but the compound showed lower inhibitory than acarbose used as a positive control (IC_50_=32.22 ± 0.40 µM).

**Table 4:** The IC50 (µM) values of the compounds on α-glucosidase.

	α-glucosidase
BDPY-2	218.62 ± 8.71
BDPY-4	94.99 ± 4.77
BDPY-5	105.83 ± 5.03
Acarbose	32.22 ± 0.40

### 3.4. DNA hydrolytic cleavage properties of the compounds

**Figure 6:**
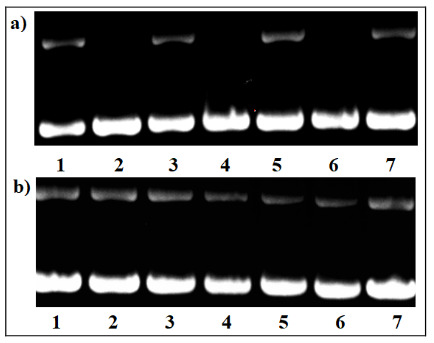
DNA hydrolytic nuclease effects of the compounds for 30 min (a), 60 min (b). Lane 1: DNA control; lanes 2–3: 25 and 50 μM of (BDPY-2 ); lanes 4-5: 25 and 50 μM of (BDPY-4 ); lanes 6-7: 25 and 50 μM of (BDPY-5 ).

The DNA hydrolytic cleavage actions of the compounds on supercoiled pBR322 plasmid DNA were determined according to our previously reported methods, and the intensity bands were observed under UV illuminator [32]. To investigate the ability of the compounds to damage the phosphodiester bonds of supercoiled plasmid DNA, we designed hydrolytic cleavage studies. The supercoiled plasmid DNA has three forms in agarose gel: form I (supercoiled form), form II (nicked form cleavage of one strand), form III (linear form cleavage of two strands). The results are presented in Figure 6. It is known that supercoiled pBR322 plasmid DNA (Thermo Fischer Scientific, SD0041) is in the supercoiled form at a rate of more than 90%. Since the plasmid DNA has impurity, the density of Form II is increased on negative controls. In this study, the presence of the compounds did not have DNA hydrolytic cleavage effects at 25 and 50 µM as compared to negative controls (Figures 6(a),(b), lanes 1) under our experimental conditions. The results claimed that these compounds may low toxicity potential in the dark as a preliminary study.

## 4. Conclusion

In conclusion, we have synthesized new monostyryl (**BDPY-2**) and distyryl BODIPY dyes (**BDPY-4, BDPY-5**) and investigated their acetylcholinesterase from *Electrophorus electricus* (AChE), butyrylcholinesterase from equine serum (BuChE), α-glucosidase from *Saccharomyces cerevisiae*, and DNA hydrolytic cleavage actions. The compounds showed varying inhibition actions against AChE, BuChE, and α-glucosidase. **BDPY-4,** which was a noncompetitive inhibitor, was the most potent compound on AChE with an IC_50_ of 54.78 ± 4.51 µM. The UV-vis spectroscopy studies claimed that it interacted with enzyme change the microenvironment around AChE. In addition, the compounds had low α-glucosidase inhibitory effects when compared to acarbose. The DNA hydrolytic cleavage was not showed on supercoiled plasmid DNA in the presence of the compounds at 25 and 50 µM as compared to negative controls under our experimental conditions. These findings suggested that these compounds have low toxicity potential in the dark. Further studies are needed regarding anticholinesterase and toxicity effects of **BDPY-4** on development of formulations, cell cultures, and in vivo studies for application in internal diseases.
